# Advances in glaucoma biomechanics from 2000 to 2024: a bibliometric study and visualization analysis

**DOI:** 10.3389/fnins.2025.1694948

**Published:** 2026-01-05

**Authors:** Tingli Wen, Xiangyu Fu, Vincentius Yonathan Evandrew, Xianjie Yu, Meng Wang, Junrong Chen, Li Tang

**Affiliations:** Department of Ophthalmology, West China Hospital, Sichuan University, Chengdu, China

**Keywords:** glaucoma, biomechanics, optic nerve head, corneal hysteresis, bibliometric, CiteSpace, VOSviewer

## Abstract

**Objectives:**

Glaucoma is an irreversible progressive optic neuropathy characterized by neurodegeneration of retinal ganglion cells. Elevated intraocular pressure induces mechanical stress and strain at the optic nerve head, impairing axonal function and contributing to the onset and progression of glaucoma. This study aims to generalize and visualize the dynamic evolution and research hotspots of glaucoma biomechanics through bibliometric methods.

**Methods:**

All documents about glaucoma biomechanics research in 2000–2024 were extracted from two databases (Web of Science Core Collection and PubMed). The hotspots and frontiers of research in terms of remarkable countries, authors, journals, keywords, and cited references were analyzed using CiteSpace and VOSviewer programs.

**Results:**

A collection of 1,655 English articles was included, with a steady increase in annual publications. 4,464 authors from 51 countries/regions were identified, led by the United States. Ethier CR was the most prolific contributor, while Downs JC was the most commonly cited author. Among 203 journals, *Investigative Ophthalmology & Visual Science* both led in publications and citations. In the domain of glaucoma biomechanics, keywords “lamina cribrosa,” “open angle glaucoma,” and “biomechanical properties” exhibited the significant centrality. The cited references formed 7 clusters, namely #0 (optic nerve head), #1 (ocular response analyzer), #2 (sclera), #3 (tonometry), #4 (lamina cribrosa), #5 (trabecular meshwork), and #6 (corneal biomechanics).

**Conclusion:**

Our study employed bibliometric and visualization analyses to state the biomechanical mechanisms underlying intraocular pressure regulation and optic nerve damage, offering the development in the field of glaucoma biomechanics. Future directions may focus on the interdisciplinary applications of artificial intelligence, tissue engineering and novel biomaterials in glaucoma pathogenesis and therapies.

## 1 Introduction

Glaucoma is an irreversible progressive optic neuropathy characterized by neurodegeneration of retinal ganglion cells (RGCs), leading to irreversible visual impairment. It is estimated that the number of people with glaucoma worldwide will increase to 111.8 million in 2040 (Tham et al., 2014). The complex pathogenesis of glaucoma is not fully certified, and decreasing intraocular pressure (IOP) is thought to be the only available therapy to prevent blindness so far, which is not always effective. For the majority of the population, the normal range of IOP is 10–21 mmHg, but the threshold for “elevated IOP” is various among different individuals (Sihota et al., 2018).

Previous studies have proposed that the IOP-related stress and strain affected axonal function at the optic nerve head (ONH), which caused local extracellular matrix (ECM) remodeling and RGCs death (Burgoyne et al., 2005). Mechanical stress and stretching can lead to the compression, deformation, and remodeling of the lamina cribrosa (LC), where the optic nerves traverse, resulting in axon damage and transport abnormalities, which is the mechanical basis of optic neuropathy in glaucoma (Quigley et al., 1983). Thus, ocular biomechanical parameters may mediate the disjointed relationship between IOP and glaucomatous optic neuropathy.

Extensive research has demonstrated that the biomechanical properties of various ocular structures, including the cornea, sclera, LC, ONH, and trabecular meshwork (TM), are closely associated with the pathogenesis and progression of glaucoma (Susanna et al., 2018; Korneva et al., 2020; Karimi et al., 2022; Kang et al., 2024). The corneal biomechanical properties such as central cornea thickness (CCT) and corneal hysteresis (CH), have impact on the accuracy of IOP measurements (Vinciguerra et al., 2020) and a potential association with the occurrence and development of glaucoma (Brazuna et al., 2023). Peripapillary sclera (PPS), scleral canal, and LC collectively constitute the connective tissue of the ONH, which is a key area of glaucoma biomechanics (Downs et al., 2005a). The abnormal structure and mechanical properties of this area may lead to compression and ischemia of optic nerve fibers, thus cause optic nerve injuries. Beyond the direct mechanical trauma to RGCs due to anatomical proximity, ocular biomechanics can also contribute to glaucomatous optic neuropathy through its impact on aqueous humor (AH) dynamics. Specifically, the elasticity and permeability of TM influences the outflow resistance of AH (Karimi et al., 2022), and iris stiffness plays a role in the narrowing and closure of the anterior chamber angle (Zhang et al., 2016; Pant et al., 2018).

Recent years have witnessed significant advancements in the biomechanical research of glaucoma. Notably, the identification of the mechanically-activated (MA) cation channel Piezo1 has emerged as a groundbreaking discovery (Rudzitis et al., 2024; Fukuoka et al., 2025). In the TM, Piezo1 can regulate IOP by responding to mechanical stress changes, causing the influx of Ca^2+^ cation, and converting mechanical stimuli into downstream cellular electrical signals (Uchida et al., 2021). Additionally, the application of novel bioengineered materials to simulate ocular structures, and the utilization of artificial intelligence (AI)-driven predictive models to investigate the relationship between ocular biomechanics and glaucoma, have also emerged as prominent research hotspots (Liou et al., 2020; Bikuna-Izagirre et al., 2024; Braeu et al., 2024).

Recently, bibliometric studies and visualization analyses have become an essential tool for biomedical research literature analysis. Bibliometric analysis can summarize and visualize the existing publications, which would assist to identify the research hotspots and evolution trends in a particular research field. Despite multiple researches on the association between glaucoma and ocular biomechanics, there is still a lack of visualized analysis and bibliometric summaries on the progression and development trends of the hotspots of glaucoma-related ocular biomechanics. Therefore, this study aims to offer a perspective into the advancement and the latest frontiers in the field of glaucoma biomechanics by bibliometric analysis.

## 2 Materials and methods

### 2.1 Search strategies and data collection

The Science Citation Index Expanded (SCIE) and Social Sciences Citation Index (SSCI) of Web of Science Core Collection (WoSCC), and PubMed database were searched for all literature on glaucoma and ocular biomechanics. All searches were completed on the same day to avoid bias in the number of documents due to database updates. Two authors completed the literature search independently by reading all titles and abstracts and skimming the full text of some ambiguous documents. The retrieved publications had to satisfy the following criteria:

1. The search terms were determined by the TS = (“glaucoma” OR “ocular hypertension” OR “intraocular pressure” OR “trabecular meshwork” OR “optic nerve head” OR “lamina cribrosa” OR “peripapillary sclera”) AND TS = (“biomechanic*” OR “ocular rigidity” OR “stiffness” OR “strain” OR “corneal hysteresis” OR “viscoelastic*” OR “finite element” OR “mechanotransduction”)
2. The document type was “Article”;
3. The language of article was “English”;
4. The publication period was from 2000-01-01 to 2024-12-31.

**Figure 1:**
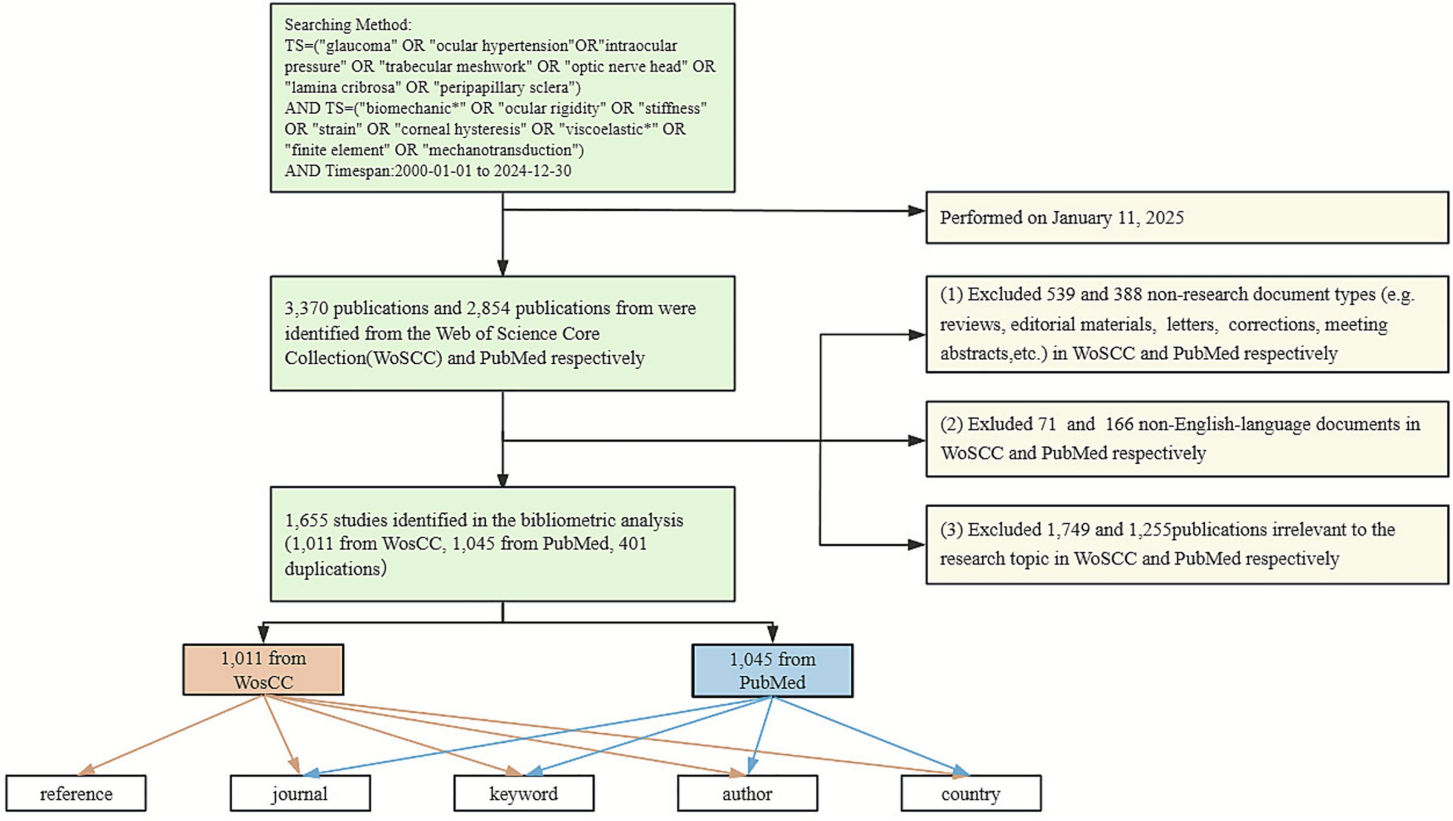
The flowchart of data collection and retrieval strategy. A total of 1,655 publications were included in the bibliometric analysis.

A total of 3,370 and 2,854 publications were initially identified from WoSCC and PubMed, respectively. Non-research document types (including reviews, editorial materials, book chapters, proceedings papers, letters, corrections, and meeting abstracts, etc.), non-English publications, and literatures irrelevant to the search topic were excluded. Finally, a total of 1,655 distinct publications (1,011 from WoSCC and 1,045 from PubMed, excluding 401 duplications) were included in this bibliometric study (Figure 1). Eligible records were saved and exported as plain text files, including titles, authors, keywords, countries, publishing journals, references, and citations.

Given the data retrieved from PubMed database have limitations in citation analysis, reference-related analyses (e.g., clusters of references, references with citation bursts) were exclusively conducted using the WoSCC database, supplemented by a systematic PubMed search to validate research development trends and ensure comprehensive data coverage and methodological rigor. For national collaboration mapping, author networks, and keyword co-occurrence analyses, we employed a merged dataset combining both WoSCC and PubMed records.

### 2.2 Bibliometric analyses

Bibliometric analysis provides a scientific and quantitative research methodology that enables researchers to identify active authors, countries, foundational knowledge, development, research hotspots, and frontiers for a specific topic. Bibliometric techniques can be mainly divided into evaluative and relational bibliometrics (Ninkov et al., 2022). Evaluative bibliometrics assess the contributions and influence of authors and countries through numerical data. Relational bibliometrics identify the themes and research hotspots via co-authorship, co-occurrence, and co-citation.

Co-authorship analysis identifies collaborative networks by tracking joint contributions of authors/countries to publications. Co-occurrence analysis reveals thematic relationships through simultaneous presence of keywords in documents, highlighting concept proximity and emerging hotspots. Co-citation analysis quantifies document associations via shared citations in reference lists, identifying the authoritative articles and foundational knowledge within a field.

In this study, the exported data from databases were imported into CiteSpace version 6.4. R1 (Drexel University, Philadelphia, United States) and VOSviewer version 1.6.20 (Leiden University, Leiden, Netherlands) to perform the bibliometric analyses. CiteSpace summarized leading countries, keywords and references with strongest citation bursts, dual journal overlay visualization, as well as create cluster and timeline maps (author and references) due to its time-slicing algorithm. VOSviewer applied to analyses of authors, journals, and co-occurrence of keywords for its strength in clustering large-scale collaboration data. Meanwhile, to facilitate the visualization of global publications, the national map was generated by SCImago Graphica version 1.0.53.

### 2.3 Calculation of total link strength between countries

Total link strength (TLS) quantifies the cumulative connection frequency between countries, where links may represent co-authorship, co-occurrence, or co-citation relationships. Higher values for specific countries indicate more extensive collaborations, which can reflect their academic influence within the research domain.

For any two nodes *i* and *j*, the formula for calculating the link weight between nodes *i* and *j* is as follows:


$$S_{\mathrm{ij}}=\frac{c_{\mathrm{ij}}}{w_{i}\times w_{j}}.\frac{w}{2}\;\mathrm{TLS}=\sum S_{\mathrm{ij}}$$

*C_ij_* represents the co-occurrence frequency of nodes *i* and *j* (e.g., country/author collaboration count, keyword co-occurrence); *w_i_* and *w_j_* are the weights of nodes *i* and *j, respectively,* (e.g., the occurrence frequency of the nodes); *W* is the total weight of all links in the network (the sum of all *C_ij_*).

## 3 Results

### 3.1 Trends of publications

**Figure 2:**
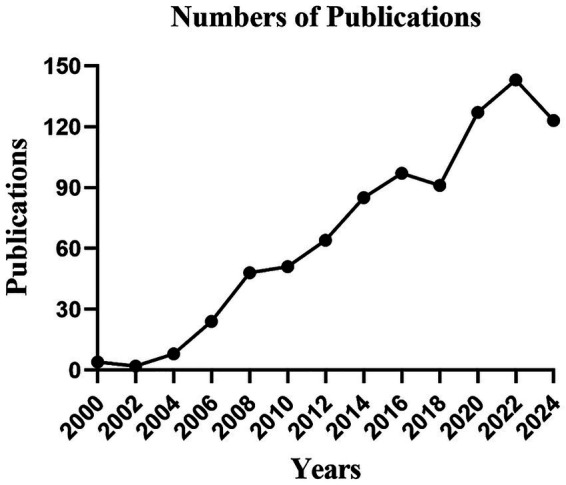
Publications of annual year. The *X*-axis represents the publish years, and the *Y*-axis represents the number of publications.

The trend changes in the number of publications reflect the degree of research attention and lucubration of knowledge about a subject. As shown in Figure 2, glaucoma biomechanics received fewer attention before 2004. However, despite some fluctuates, the number of published articles shows a steady upward trend from 2004 to 2024. The result indicates that the ocular biomechanics in glaucoma has received more attention and become a research hotspot in recent years.

### 3.2 Analysis of leading countries

**Figure 3:**
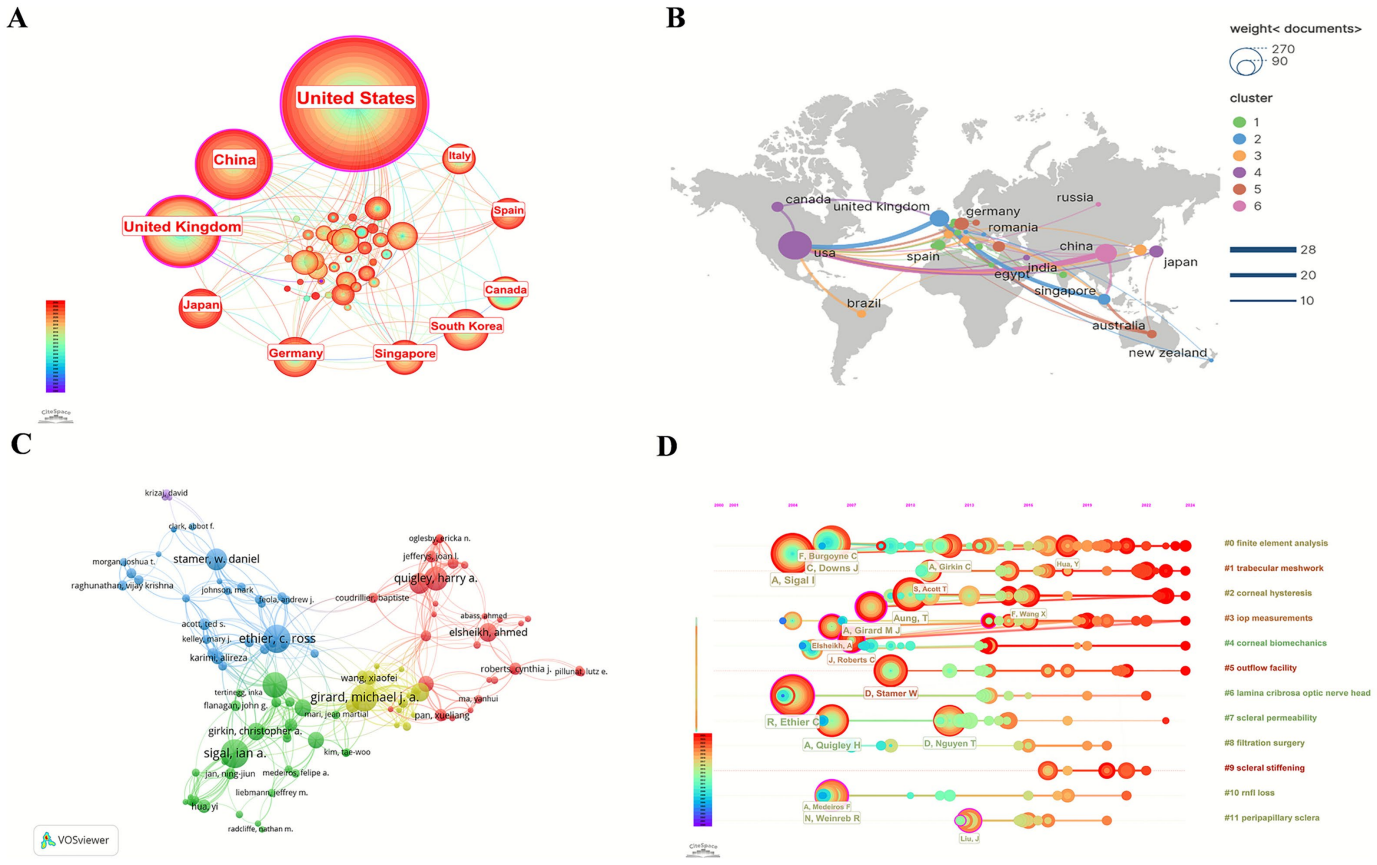
The bibliometric analysis of countries and authors. Each node represents an individual country/author, and the node size is proportional to the number of publications. Line thickness between nodes indicates link strength of a collaboration relationship. Different colors reveal various clusters. **(A)** Cooperation map of countries/regions. **(B)** Distribution and collaboration of countries/regions. **(C)** The network of authors of glaucoma biomechanics. The node label presents the author’s name and the node size represents the co-citation frequency. **(D)** Timeline views of clusters of top authors in the field of glaucoma biomechanics. The *X*-axis represents the year, and the *Y*-axis displays the name of each cluster.

Relevant materials were published in 51 countries/regions. Table 1 displays the 10 countries with the most publications. The publications of the United States (USA) and China accounted for more than half of the total. According to the citation frequency analysis, USA published 566 articles, with the highest citations (23,352), ranking first among all countries, followed by China and the United Kingdom (UK). The geographical distribution of countries engaged in glaucoma biomechanics shows extensive global participation and robust collaborative networks, as visually summarized in Figures 3A,B.

**Table 1:** Top 10 country distributions of publications.

Rank	Country	Counts	Citations	Total link strength
1	United States	566	23,352	241
2	China	167	3,394	120
3	United Kingdom	154	8,438	193
4	Japan	64	1,075	21
5	Germany	63	2,667	48
6	Singapore	58	2,230	112
7	South Korea	49	1,251	33
8	Canada	40	2,818	27
9	Spain	36	553	17
10	Italy	35	1796	43

Total link strength: the cumulative connection frequency between countries, higher values signify more extensive international collaborations.

### 3.3 Analysis of authors and cited authors

Overall, 4,464 researchers participated in the glaucoma biomechanics studies. Table 2 shows the top 10 productive authors, cited author in WoSCC. Table 2 demonstrates that the research field is predominantly led by USA scholars, while also featuring international participation to some extent. Ethier CR from USA, with 92 publications, emerges as the most prolific contributor, followed by Sigal IA from USA (80 publications) and Girard MJA from Singapore (79 publications). The cited author analysis shows Downs JC and Ethier CR top the list with 3,117 and 2,888 citations, respectively.

**Table 2:** The top 10 authors and cited authors.

Rank	Author	Country	Counts	Cited author	Country	Citations
1	Ethier CR	United States	92	Downs JC	United States	3,117
2	Sigal IA	United States	80	Ethier CR	United States	2,888
3	Girard MJA	Singapore	79	Burgoyne CF	United States	2,882
4	Downs JC	United States	65	Sigal IA	United States	2,643
5	Stamer WD	United States	63	Girard MJA	Singapore	2,596
6	Liu J	United States	48	Quigley HA	United States	2080
7	Quigley HA	United States	45	Nguyen TD	United States	1877
8	Aung T	Singapore	44	Elsheikh A	United Kingdom	1822
9	Nguyen TD	United States	40	Weinreb RN	United States	1,532
10	Burgoyne CF	United States	39	Stamer WD	United States	1,412

Figures 3C,D reveal the author collaboration network and timeline analysis of top authors clusters, respectively. Analysis of authors presents that 60 out of 4,464 authors had publication volumes of at least 15 (Figure 3C). The network was divided into 4 main clusters represented by different colors. The four distinct clusters (blue, green, red, and yellow) are, respectively, centered around Ethier CR, Sigal IA, Quigley HA, and Girard MJA, with interconnecting lines demonstrating collaborative relationships among authors. The timeline analysis of top author clusters reveals the dominant research themes and active periods of leading scholars in some extent, with colors representing publication years as annotated in the left color scale (Figure 3D). As seen in the Figure 3D, Sigal IA has conducted deep research in “finite element analysis” of glaucoma biomechanics since 2004 and remains actively engaged in this domain.

### 3.4 Analysis of journals and cited journals

The retrieved articles on glaucoma biomechanics were published in 203 journals. Table 3 lists the top 10 productive journals and co-cited journals in WoSCC. The publications of top 10 productive journals account for 44.5% (736/1,655) of the total publications. *Investigative Ophthalmology & Visual Science* (*IOVS*) emerges as the dominant journal with 270 publications, followed by *Experimental Eye Research* with 104 publications and *Journal of Glaucoma* with 90 publications. Meanwhile, the appearance of bioengineering journals such as *Acta Biomaterialia* and *Journal of Biomechanical Engineering-transactions of the Asme* also indicates the interdisciplinary nature of the research.

**Table 3:** The top 10 journals and cited journals.

Rank	Journal	Counts	Cited journal	Citations
1	*Investigative Ophthalmology & Visual Science*	270	*Investigative Ophthalmology & Visual Science*	10,866
2	*Experimental Eye Research*	104	*Experimental Eye Research*	2,599
3	*Journal of Glaucoma*	90	*Journal of Glaucoma*	1,594
4	*Plos One*	48	*Ophthalmology*	1,493
5	*Scientific Reports*	43	*American Journal of Ophthalmology*	1,461
6	*American Journal of Ophthalmology*	41	*British Journal of Ophthalmology*	1,026
7	*Journal of Biomechanical Engineering-transactions of The Asme*	39	*Journal of Biomechanical Engineering-transactions of The Asme*	1,014
8	*Acta Biomaterialia*	35	*Plos One*	993
9	*British Journal of Ophthalmology*	33	*Biomechanics and Modeling in Mechanobiology*	798
10	*Current Eye Research*	33	*Acta Ophthalmologica*	737

Citation analysis of journals reflects the influence of journals in the field of glaucoma biomechanics. *IOVS* leads the list of cited journals with a substantial citation counts of 10,866, indicating its pivotal role in disseminating high-impact research. *Experimental Eye Research* ranked second with 2,599 citations, followed by *Journal of Glaucoma* (1,594) and *Ophthalmology* (1,493), reflecting their superior quality of the publications and significant academic influence within the field.

**Figure 4:**
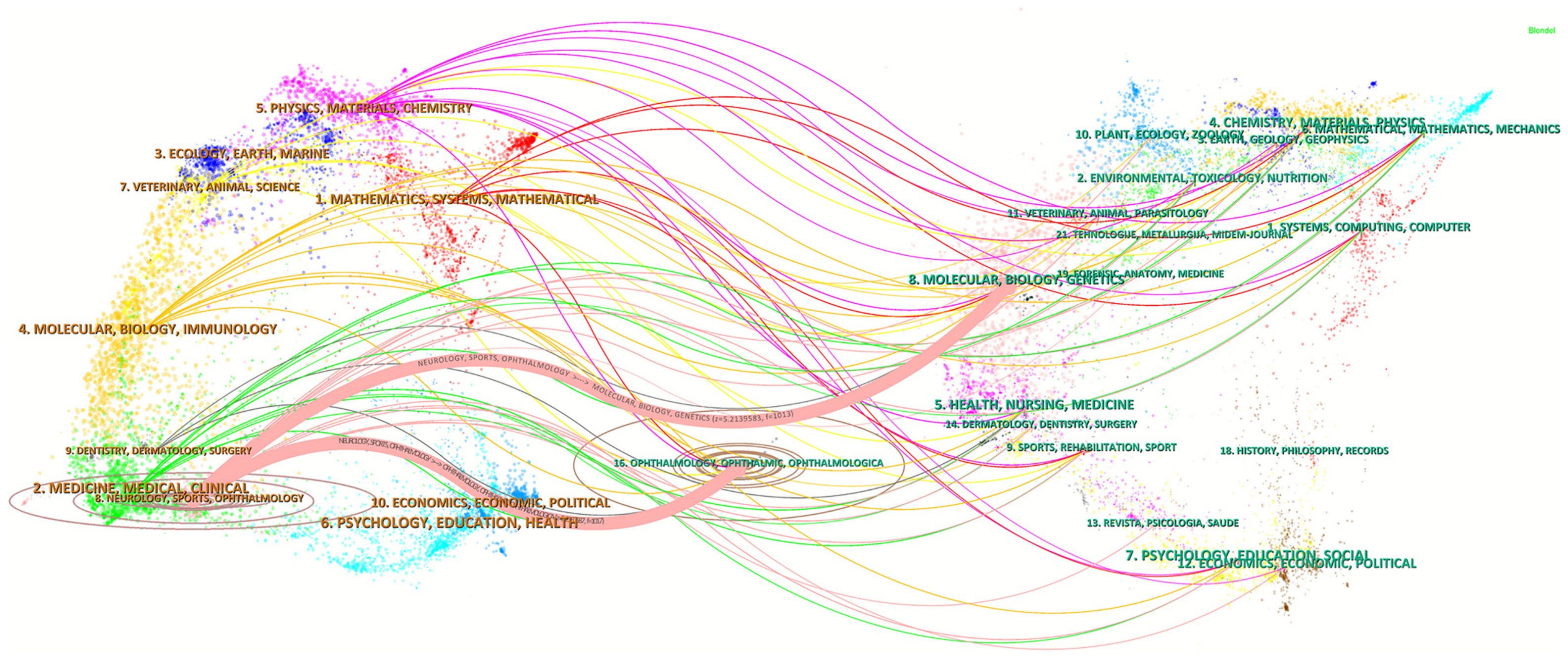
The dual-map overlay of journal, left side represents citing publications, while the right side displays cited references. Lines connecting from left to right indicate the evolution of research focus within this field.

Furthermore, through the dual journal overlay visualization, the intersection and development of research fields can be reflected by the changes in journals. In Figure 4, the left side represents citing journals while the right side indicates cited journals, and the connecting pathways denote citation flow direction. Two primary citation tracks both originated from Neurology/Sports/Ophthalmology and terminated at Molecular/Biology/Genetics and Ophthalmology/Ophthalmic/Ophthalmologica, respectively, underscoring the multidisciplinary integration of glaucoma biomechanics with optic nerve biology, molecular, genetics, and biomechanical engineering.

### 3.5 Analysis of co-occurring keywords and burst terms

**Figure 5:**
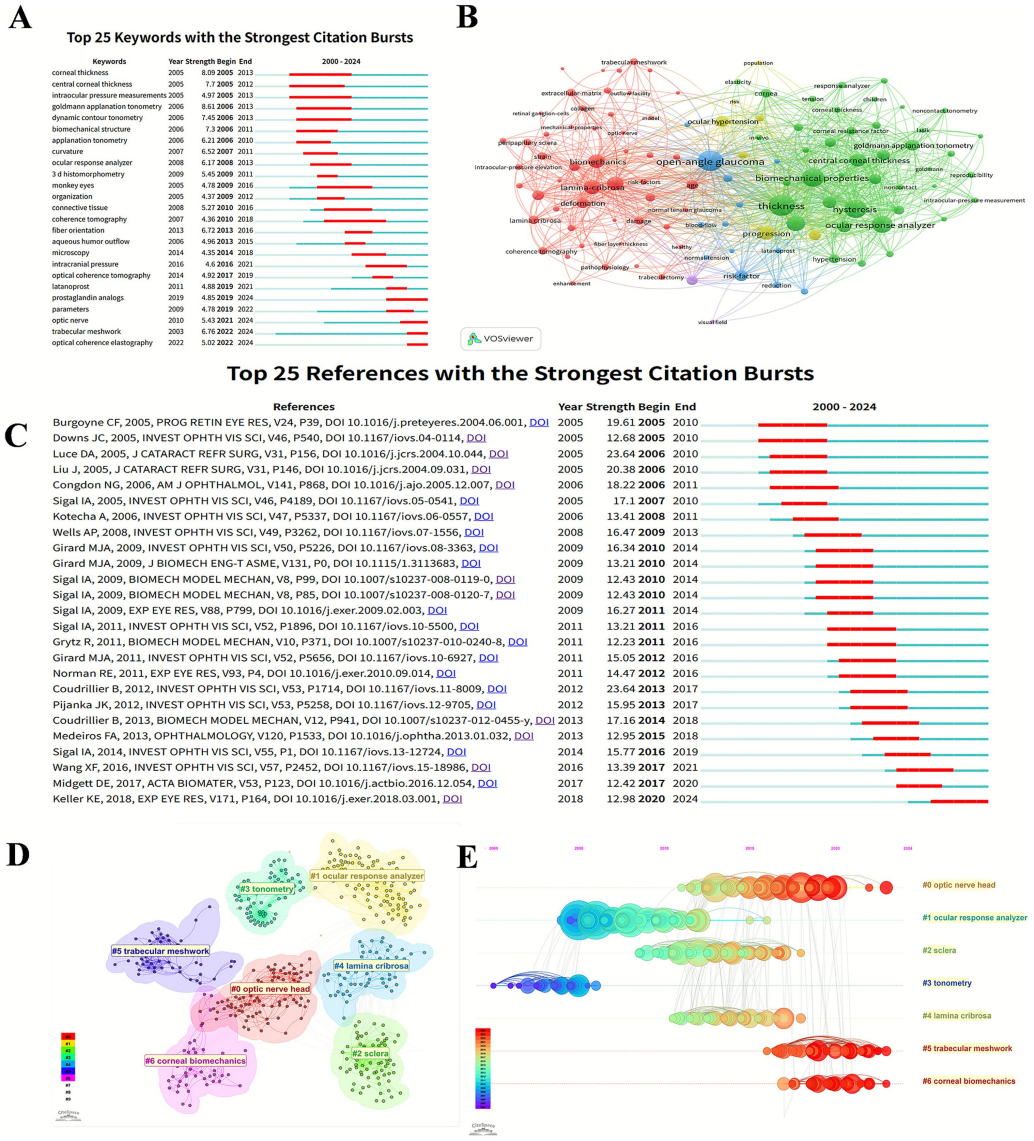
The bibliometric analysis of keywords and references. **(A)** Top 25 keywords with the strongest citation bursts in glaucoma biomechanics. **(B)** The co-occurrence analysis of keywords. The size of each node refers to the frequency of the keywords, and the links connecting two nodes represent a co-occurrence relationship between two keywords. **(C)** Top 25 references with the strongest citation bursts in glaucoma biomechanics. **(D)** Citespace visualization clusters of co-citations in glaucoma biomechanics. **(E)** Timeline views of clusters of co-cited references about glaucoma biomechanics studies. The *X*-axis represents the year, and the *Y*-axis displays the name of each cluster.

The analysis of keywords can demonstrate the hotspots and focus of this research field. As shown in the Figure 5A, the evolution of keywords with the strongest citation bursts can reflect the evolutionary process of glaucoma biomechanics research. From 2000 to 2024, the technological evolution in glaucoma biomechanics assessment has progressed from the “goldmann applanation tonometry,” “dynamic contour tonometry” to “ocular response analyzer” and “optical coherence tomography,” while research focus has shifted from foundational ocular parameters (e.g., central corneal thickness) toward microscopic concepts such as “fiber orientation” and “trabecular meshwork.” Furthermore, recent years have also witnessed emerging research on the mechanistic role of “intracranial pressure” in the pathogenesis of glaucoma and the effect of anti-glaucoma medications such as “prostaglandin analogs” on the biological characteristics (Martinez-Sánchez et al., 2023; Shen et al., 2023; Zhu et al., 2023).

VOSviewer extracted and clustered the keywords with a minimum of 10 occurrences. Figure 5B shows the keyword co-occurrence visualization network, which can be divided into 3 main clusters represented by different colors. The red category mainly concerns ocular anatomical structures, such as “lamina-cribrosa,” “trabecular meshwork,” and “peripapillary sclera.” The blue cluster involves ocular diseases like “open-angle glaucoma,” “ocular hypertension,” and “normal tension glaucoma.” The green cluster is mostly related to ocular biomechanical properties including “thickness,” “hysteresis,” and “elasticity.”

### 3.6 Analysis of cited references

#### 3.6.1 Top cited references

Highly cited references can efficiently reveal the knowledge foundation and progression of the research field. Figure 5C displays top 25 references with citation bursts, which reveals the thematic progression in glaucoma biomechanics.

The earliest reference with a citation burst is the study by Burgoyne CF, in which he reconceptualized the ONH as a biomechanical structure and proposed mechanistic hypothesis for axonal damage in glaucoma (Burgoyne et al., 2005). Subsequent research have addressed glaucomatous alterations in ocular biomechanical properties, including peripapillary sclera (Downs et al., 2005b), and corneal biomechanics (Luce, 2005), while the latest studies have transitioned toward more microscopic biomechanical phenomena, such as the associations between eye movement patterns and ONH mechanical strain (Wang et al., 2016), and the regional strain variation in LC (Midgett et al., 2017).

Table 4 lists the top 10 highest cited references in the field of glaucoma biomechanics in WoSCC. Sigal IA’ s publication was published in *IOVS* in 2005 and received the most citations (430). Among the ten publications, four papers established computational models targeting the ONH or TM to predict the risk of glaucoma progression (Bellezza et al., 2000; Sigal et al., 2004a; Sigal et al., 2005a; Last et al., 2011); three articles focused on the relationship between scleral biomechanical properties and glaucoma (Girard et al., 2011b; Coudrillier et al., 2012; Vurgese et al., 2012); the other three publications evaluated specific biomechanical properties using different measurements (Congdon et al., 2006a; Kotecha et al., 2006; Medeiros and Weinreb, 2006), among which CH and CCT were the most concerned.

**Table 4:** The top 10 highest cited articles.

Rank	Title	Author	Year	Citations	Main content
1	Factors influencing optic nerve head biomechanics.	Sigal IA et al.	2005	430	Measured the influence of ocular tissue biomechanical properties on ONH biomechanical response for acute IOP changes by using computational model (Sigal et al., 2005a).
2	Central corneal thickness and corneal hysteresis associated with glaucoma damage	Congdon NG et al.	2006	403	Investigated the impact of CCT and CH on glaucoma damage (Congdon et al., 2006a).
3	Corneal thickness-and age-related biomechanical properties of the cornea measured with the ocular response analyzer	Kotecha A et al.	2006	353	Assessed CH/CCT impact to IOP and compared ORA-GAT agreement between patients with ocular hypertension and the normal population (Kotecha et al., 2006).
4	Elastic modulus determination of normal and glaucomatous human trabecular meshwork.	Last JA et al.	2011	297	Developed a model to compare the elastic modulus of trabecular meshwork in glaucoma and normal eyes and predicted the impact to AH outflow facility (Last et al., 2011).
5	Biomechanics of the human posterior sclera: age- and glaucoma-related changes measured using inflation testing.	Coudrillier B et al.	2012	287	Measured the biomechanical response of the human posterior sclera *in vitro* and predicted a stiffer response in peripapillary sclera of glaucoma eyes (Coudrillier et al., 2012).
6	Finite element modeling of optic nerve head biomechanics.	Sigal IA et al.	2004	258	Characterized the biomechanics of the optic nerve head by computer modeling (Sigal et al., 2004a).
7	The optic nerve head as a biomechanical structure: initial finite element modeling	Bellezza AJ et al.	2000	253	Analyzed the relationship between the stress within the load-bearing connective tissues of the optic nerve head and IOP by finite element model (Bellezza et al., 2000).
8	Biomechanical changes in the sclera of monkey eyes exposed to chronic IOP elevations.	Girard MJ et al.	2011	243	Characterized scleral biomechanics under chronic IOP elevation in monkey eyes (Girard et al., 2011b).
9	Evaluation of the influence of corneal biomechanical properties on intraocular pressure measurements using the ocular response analyzer.	Medeiros FA et al.	2006	235	Evaluated the relationship between corneal biomechanical properties and IOP measured by ORA and GAT (Medeiros and Weinreb, 2006).
10	Scleral thickness in human eyes	Vurgese S et al.	2012	233	Measured scleral thickness in different ocular regions and its correlation with glaucoma (Vurgese et al., 2012).

ONH, optic nerve head; IOP, intraocular pressure; CH, corneal hysteresis; CCT, corneal central thickness; ORA, ocular response analyzer; GAT, goldmann applanation tonometry; AH, aqueous humor.

#### 3.6.2 Seven clusters of the co-citation network

Citespace can split the co-cited references into several clusters, exhibiting closely associated publications in the same cluster and vaguely associated ones in separate clusters. The definition of each cluster derives from the most frequent keyword terms of the citing papers in this cluster. As shown in Figure 5D, there are 7 clusters differentiated by various colors in a visualization network map, which are #0 optic nerve head, #1 ocular response analyzer, #2 sclera, #3 tonometry, #4 lamina cribrosa, #5 trabecular meshwork, and #6 corneal biomechanics, respectively. Five (#0, #2, #4, #5, and #6) of these clusters are about ocular tissue biomechanical properties, whereas the other two (#1, #3) focus on the biomechanical measurements.

The cluster map (Figure 5D) could be turned into a timeline view (Figure 5E), which describes the general progression of seven clusters over time. Clusters #1 (ocular response analyzer) and #3 (tonometry) appeared in an earlier period, while cluster #0 (optic nerve head), #5 (trabecular meshwork), and #6 (corneal biomechanics) persisted to recent times.

### 3.7 Research trends in PubMed

Using CiteSpace and VOSviewer, a bibliometric analysis was conducted on 1,045 publications retrieved from PubMed. Through the analysis of keyword frequence, the research trends in the field of glaucoma biomechanics were generally grasped (Table 5). Among these keywords, research themes such as “intraocular pressure,” “glaucoma” and “biomechanical phenomena” appeared with the highest frequencies. Moreover, methodological keywords including “finite element analysis” and “biological model” underscore increasing multidisciplinary collaboration with computational science and tissue engineering.

**Table 5:** The top 15 most frequently occurring keywords in PubMed.

Rank	Keyword	Frequence
1	intraocular pressure	654
2	glaucoma	644
3	biomechanical phenomena	387
4	cornea	366
6	middle aged	305
7	tonometry	282
8	optic disk	224
9	sclera	204
10	elasticity	181
11	trabecular meshwork	138
12	optical coherence tomography	134
13	prospective studies	108
14	finite element analysis	105
15	biological model	101

## 4 Discussion

In the field of ophthalmology, the relation between biomechanics and glaucoma has been a study subject for many years and has been further explored continuously (Figure 2). Bibliometric analysis has emerged as a prominent methodology for identifying active research hotspots and forecasting future trends through the generation of visualized maps, which is widely recognized as a valuable tool for mining useful information from the complex network structure of literature data (Ninkov et al., 2022). In this study, we conducted a bibliometric analysis of glaucoma biomechanics, and identified publication trends and global contributions through visualized maps. To the best of our knowledge, this study is the first bibliometric study and visualization analysis in this field.

### 4.1 Global trends in glaucoma biomechanics research

In total, 4,464 authors from 1,020 institutions in 51 countries/regions have published articles in 203 journals on this subject, which can demonstrate the global collaboration in the field of glaucoma biomechanics and the widespread attention from the scientific community.

As shown in Table 1 and Figure 3A,B, there was extensive cooperation among countries. USA was the most productive country with the highest citations, which was obviously the core of study collaboration, followed by China and UK. Meanwhile, the distribution of top 10 productive authors and cited authors underscores the sustained leadership of USA researchers in advancing glaucoma biomechanics studies. Furthermore, the presence of Girard MJA and Aung T from Singapore in the list have demonstrated the global dissemination of glaucoma biomechanics research (Table 2).

### 4.2 Development in glaucoma biomechanics

The clusters of references can reveal the research trends and hotspots in glaucoma biomechanics, including #0 optic nerve head, #1 ocular response analyzer, #2 sclera, #3 tonometry, #4 lamina cribrosa, #5 trabecular meshwork, and #6 corneal biomechanics (Figure 5E). Here, we have systematically synthesized the development of glaucoma biomechanics through a thematic analysis of clustered research domains.

#### 4.2.1 Ocular response analyzer, tonometry, and corneal biomechanics (#1, #3, #6)

As the outermost layer of the eyeball, the cornea serves as the primary structure for detecting the response to mechanical stress. Its biomechanical properties are the most accessible and clinically relevant parameters. Traditional tonometers like non-contact tonometer (NCT) calculates IOP indirectly through measurement of air jet resistance forces by directing a pulsed air stream toward the central cornea, while Goldmann applanation tonometry (GAT), which recognized as the clinical gold standard, measures the force required to flatten a predefined corneal area. However, the IOP measured by those methodologies is closely associated with characteristics of the cornea, including CCT, rigidity, and viscosity. With the emergence of new equipment including Ocular Response Analyzer (ORA), Corvis ST, and Dynamic contour tonometer, etc., it was feasible to measure biomechanical properties of the cornea *in vivo* (Boszczyk et al., 2022; Pniakowska et al., 2022; Qu et al., 2024). ORA employs dynamic bidirectional applanation technology, wherein an air jet similar to that used in NCT is used to cause a first inward applanation and then a second outward rebound (Chong and Dupps, 2021). Through quantitative analysis of temporal disparities, it derives biomechanical parameters including CH and corneal-compensated IOP (IOPcc). Corvis ST uses an ultra-high-speed scheimpflug camera (4,330 frames/s) to capture dynamic corneal deformation responses to air-puff stimulation, enabling derivation of biomechanical parameters including stiffness parameters(SPs) and calculation of biomechanically corrected IOP (bIOP) (Boszczyk et al., 2022). Some studies suggested that IOPcc or bIOP seem to provide an estimate of IOP which is less influenced by corneal properties than those provided by GAT, especially in those with cornea disease or refractive surgeries (Luce, 2005; Medeiros and Weinreb, 2006; Bao et al., 2020; Salouti et al., 2023).

Meanwhile, the association between corneal biomechanics parameters including CCT, CH, SPs and glaucoma has quickly become a hot spot in glaucoma biomechanics research. Due to the fact that both the corneal stroma and sclera originate from the mesoderm and are directly connected, the corneal biomechanical behaviors can not only directly influence IOP measurement accuracy, but also can partly reflect the biomechanical properties of the sclera and even the degree of glaucomatous optic nerve damage (Laroche et al., 2022). CCT was the first parameter to attract significant attention in glaucoma biomechanics research, it was recognized to be associated with the incidence of glaucoma started in 2002 when study first reported that a thin central cornea is an independent risk factor for the conversion of ocular hypertension to open-angle glaucoma (Gordon et al., 2002). Quigley HA found that thinner CCT was associated with the state of glaucoma damage and CH were associated with progressive visual field worsening (Congdon et al., 2006b).

Qassim et al. (2021) found that glaucoma suspect eyes tend to have cornea with higher SPs and lower CCT, suggesting thin and stiff corneas are at greater risk of progression. CH is a relatively novel biomechanical property which measures viscoelasticity and represents the overall deformability of cornea. Numerous research findings suggest that CH is associated with the degree of glaucomatous damage and progression. Congdon et al. (2006b) found that lower CH is independently associated with features of glaucoma damage and related to the progression of visual field loss, suggesting that impaired corneal energy dissipation capacity may exacerbate ONH deformation under elevated IOP. Furthermore, CH is not merely a static element in the pathophysiology of glaucoma, but also a dynamic risk factor that changes with the disease and with its treatment. Studies found CH increases with treatment including topical prostaglandins and anti-glaucoma surgery, and decreases with progression in glaucoma (Agarwal et al., 2012; Hussnain et al., 2015; Pillunat et al., 2017). Meanwhile, Kaderli et al. (2020) reported the increase of CH was related to the type of surgery, Ahmed glaucoma valve implantation offers better corneal biomechanical results than standard trabeculectomy in 6-month follow-up. Therefore, postoperative higher values of corneal biomechanic parameters may offer better prognosis.

Corneal biomechanical parameters also provide certain indications in the diagnosis of normal-tension glaucoma (NTG). Comparative analyses revealed that NTG patients exhibit lower corneal stiffness compared to ocular hypertensive and healthy control groups (Liu et al., 2023), suggesting greater ocular compliance, which may explain the susceptibility of lower IOP in NTG. Therefore, axial elongation may serve as one of the risk factors for NTG (Chen et al., 2022). Additionally, Pillunat et.al introduced the Dresden biomechanical glaucoma factor, which weighs bIOP and pachymetry data alongside Corvis ST-derived metrics of corneal biomechanics to improve early detection of NTG (Pillunat et al., 2019).

#### 4.2.2 Sclera (#2)

Sclera is the main ocular load-bearing connective tissue, it affects the transmission of IOP and provides stable mechanical support to internal ocular structures such as ONH. PPS, scleral canal and LC collectively form the connective tissue of the ONH, which is the key area of glaucoma biomechanics (Downs et al., 2005a). Numerous studies showed that ONH biomechanics are strongly dependent on scleral biomechanical properties (Sigal et al., 2005b; Norman et al., 2011). Furthermore, research demonstrated that the local anisotropic arrangement of collagen fibers in the PPS plays a protective role in resisting scleral canal expansion and LC deformation (Coudrillier et al., 2013), suggesting interindividual variations in scleral properties may be a risk factor of the development of glaucoma and could explain differential susceptibility to ocular hypertension to some extent. Conversely, scleral sclerosis may also be a consequence of glaucoma. Downs et al. (2005a) have observed PPS presented stiffer pressure-strain response in uniaxial stress relaxation and tensile tests with elevated IOP. Significant thinning and stiffness of PPS follows exposure to moderate IOP elevations was found in monkey eyes, which are likely to be the result of scleral ECM remodeling (Girard et al., 2011a; Jonas et al., 2011). Meanwhile, sclera biomechanics are also closely related to high myopic associated glaucoma. It is well known that high myopia is predisposed to be complicated with glaucoma (Zhang et al., 2024), and scleral thinning caused by high myopia may lead to the increasing sensitivity of ONH to elevated IOP (Shen et al., 2016; Grytz et al., 2020).

Based on the impact of sclera biomechanics to the pathogenesis of glaucoma, the new therapy targeting sclera has been proposed (Quigley and Cone, 2013). Making sclera have a more elastic and less stiff response to mechanical stress is the main goal. In this case, scleral crosslinking may be available. Local scleral crosslinking to PPS region was reported to be beneficial by reducing the magnitude of biomechanical strains and consequently may be positive in glaucoma axonal neuroprotection (Coudrillier et al., 2016). Additionally, topical prostaglandin can increase sclera permeability thus improving uveoscleral outflow, but with as yet unknown effects on scleral mechanical behaviors.

#### 4.2.3 Trabecular meshwork (#5)

TM is directly connected to the Schlemm’s canal (SC) inner wall endothelium through juxtacanalicular tissue, together form the first resistance site of AH outflow pathway which plays an important role in the regulation of IOP. Changes in biomechanical properties of these tissues have impact on the drainage of AH. SC and TM cells can respond to mechanical stimulation by secreting factors that modulate outflow resistance, including matrix metalloproteases (Bradley et al., 2001), nitric oxide (NO) (Ashpole et al., 2014) and vascular endothelial growth factor (Reina-Torres et al., 2017). Thus, those factors lead to ECM remodeling and able to induce stiffening of AH outflow pathway. As previous studies reported, the observation of increased TM stiffness during pathologic IOP elevation supports the theory (Raghunathan et al., 2018; Li et al., 2019; Vahabikashi et al., 2019).

Li et al. (2024) reported ECM sclerosis induces pathological contraction of SC cells and ECM remodeling by activating the Yes-associated protein (YAP)/PDZ-binding motif (TAZ) signaling, resulting in increased IOP. Inactivation of YAP/TAZ pathway can attenuate TM cell pathobiology and improve AH outflow (Yemanyi and Raghunathan, 2020; Yoo et al., 2023), may be one of promising therapies for glaucoma. Meanwhile, reconstruction of TM based on new biomaterials and 3D printing technology is also one of the future research directions of TM biomechanics (Li et al., 2021; Bikuna-Izagirre et al., 2024; Adhikari et al., 2025).

#### 4.2.4 Optic nerve head and lamina cribrosa (#0, #4)

The axons of RGCs converge toward the ONH, passing through LC to exit the eyeball, where are the core regions of pathological changes in glaucoma. LC is a porous, multilayered structure composed of collagen and elastic fibers, which provides channels for the axons of RGCs to exit the eye and converge into the optic nerve, enabling the transmission of neural signals to the brain. Quigley HA et.al have observed the posterior bowing of LC in glaucoma patients through electron microscopy, proposing the biomechanical hypothesis that elevated IOP induces LC displacement and subsequent axonal compression, leading to glaucomatous mechanical damage of optic nerve (Quigley et al., 1983).

Previously, the assessment of optic nerve damage has primarily relied on the evaluation of the cup-to-disc ratio, an indirect and subjective measure that provides limited insights into the structural and biomechanical alterations. Recent advancements in imaging have progressed to directly characterize the biological properties of the ONH and LC. Lee et.al have utilized enhanced depth imaging spectral-domain optical coherence tomography (OCT) to measure LC thickness in glaucoma patients and healthy controls, revealing that the LC is significantly thinner in glaucoma eyes (Lee et al., 2011). The results are consistent with biomechanical hypotheses, suggesting that a thinner LC renders the optic nerve more susceptible to compression, acting not only as a risk factor for glaucoma but also potentially as a consequence of glaucomatous damage. Furthermore, studies have reported a significant correlation between LC thickness and visual field defects (Inoue et al., 2009), indicating that LC thickness may serve as a biomarker for assessing the severity of glaucoma.

Numerous studies have also employed computational modeling to evaluate the biomechanical properties of the ONH and LC. Among them, Sigal IA and his team has been pioneering contributors in this domain. First, they characterized the ONH biomechanics through computer modeling and proposed that strains in LC are more dependent on scleral properties (Sigal et al., 2004b). Subsequently, they used 3D histology and finite-element (FE) models to reconstruct the anatomy of the human ONH and explore the influence of tissue properties on ONH biomechanics, respectively (Sigal et al., 2009,^2010^). Building on this, their FE analysis of PPS collagen architecture revealed that tangential fiber arrangements provide superior mechanical support to scleral canal tissues compared to circular configurations (Voorhees et al., 2018). Recently, the team innovatively proposed fibrous finite element modeling (Islam et al., 2024), which concept emphasized the inhomogeneity and micro-biomechanics of the fibrous collagen structure in the ONH. Additionally, their team have also proposed the combination of the digital volume correlation method and OCT technology to characterize the in-vivo ONH deformation (Zhong et al., 2022).

Meanwhile, the forces acting on the ONH environment are also influential factors in modulating its biomechanical alterations. The forces acting on the ONH can be divided into two directions. On the one hand, the IOP exerts a direct force at the vitreoretinal interface to enlarge the eye globe and the tension in the scleral wall which is transmitted to the ONH and posteriorly displace ONH tissues. On the other hand, the elevated intracranial pressure (ICP) causes a retrolaminar tissue pressure that anteriorly displaces the lamina cribrosa. Thus, ICP acts as counter pressure across the LC that compensates IOP and relatively high ICP can compensate for elevated IOP and reduce RGC loss (Kharmyssov et al., 2019). Recent studies have recognized greater translaminar pressure difference (TPD) may participate in glaucomatous damage, especially in NTG (Stoskuviene et al., 2023; Wu et al., 2024). The increase of TPD initially elevates strain within the LC, and once the strain exceeds a critical threshold, it triggers the production of ECM, leading to fibrosis and increased stiffness of the LC (Hopkins et al., 2020). However, further studies are needed to analyze the involvement of TPD in NTG pathogenesis and management due to the heterogeneity of ICP distribution in the cerebrospinal fluid space (Pircher et al., 2017).

#### 4.2.5 Future hotspots

The future research hotspots of glaucoma biomechanics may focus on specific mechanical stress regulation channels and interdisciplinary cooperation.

Recently, the studies of MA cation channels including Piezo1 and transient receptor potential vanilloid 4 (TRPV4), have shed light on pressure-sensitive biological processes. Piezo1 was found to be required for TM mechanotransduction and increase AH outflow (Yarishkin et al., 2021). Immunostaining analysis showed that the Piezo1 were expressed in ocular tissues including TM, RGC layer, etc., and its agonist Yoda 1 suppressed neurite outgrowth in RGC (Morozumi et al., 2020). Additionally, TRPV4 channels was reported to activate endothelial nitric oxide synthase (eNOS) and product NO in TM cells, may participate in the outflow of AH and reduction of IOP (Patel et al., 2021). The functional impairment of TRPV4-eNOS signaling in glaucoma rendering TM cells insensitive to fluid flow–induced shear stress and result in the pathological elevation of IOP. Taken together, although the specific mechanisms of Piezo1-mediated glaucoma pathogenesis and TRPV4-eNOS pathway remain unclear at the molecular level, it suggests potential for novel therapeutic target for glaucoma.

As for multidisciplinary cooperation, the study of glaucoma biomechanics has transcended the traditional boundaries of ophthalmology, establishing interdisciplinary connections with materials science, artificial intelligence, and tissue engineering etc. In materials science, glaucoma biomechanics can combine with the development of advanced biomimetic scaffolds and biomechanical responsive materials for trabecular meshwork reconstruction. For example, Li et al. (2021) developed a biomimetic human trabecular meshwork (HTM) hydrogel model for investigation of 3D cell-ECM interactions under normal and simulated glaucomatous conditions, which can imitate the HTM biomechanics *in vitro*. Bikuna-Izagirre et al. (2024) used melt electrowriting and solution electrospinning to reconstruct the artificial trabecular meshwork. Future research could focus on in vitro glaucoma model experiments for bionic ocular structures.

In AI, the integration of glaucoma biomechanics with AI models holds significant promise for the diagnosis and progression prediction of glaucoma. A study of Chuangsuwanich et al. (2025) employed a geometric deep learning model integrated ONH biomechanical data, which can significantly improve the accuracy of visual field loss predictions. Braeu et al. (2024) proposed an AI-driven approach which can assess the robustness of ONH by OCT volume scan instead of traditional biomechanical testing.

In tissue engineering, collagen cross-linking can be employed to modulate the biomechanical properties of ocular tissues, thereby enhancing their resistance to damage induced by elevated IOP. For instance, Campbell et al. (2017) used collagen cross-linking agents and successfully enhanced scleral stiffness. However, the efficacy of scleral collagen cross-linking alone remains inconclusive. Studies have also reported that collagen cross-linking in sclera may increase susceptibility to RGC damage in mice (Kimball et al., 2014). Lysyl oxidase-like 1 (LOXL1) is a copper-dependent amine oxidase that maintains the structural integrity of the ECM thus form the proper elastic fiber by catalyzing the cross-linking of collagen and elastin. Recent studies have suggested that *Loxl1^−/−^* mice have significant anterior segment biometric abnormalities including the changes of elastic fibers and collagen fibrils in PPS (Wareham et al., 2022), which provide a novel animal model that can be utilized for studying scleral biomechanics. Although the specific pathways have not yet been fully elucidated, molecular interventions targeting ocular collagen remodeling including LOXL1 are still expected to become one of potential targets for the treatment of glaucoma in the future (Rahman et al., 2020).

### 4.3 Limitations

There are still several limitations. First, our analyses primarily rely on the data obtained from WoSCC. Although WoSCC is the most commonly used and authoritative comprehensive database, which can represent a significant amount of information, and publication trends from PubMed also validate our conclusions, some publication bias is still unavoidable. Second, due to the difficulty in encompassing all terms related to glaucoma biomechanics within the search query, it may have led to certain limitations and biases in the retrieved literature. Last, it should be noted that the algorithms of CiteSpace and VOSviewer may sometimes exhibit certain deviations, such as potential biases in clustering accuracy due to parameter sensitivity.

## 5 Conclusion

The field of glaucoma biomechanics is undergoing a shift from the description of macroscopic mechanical phenomena to the precise manipulation at the molecular or novel multidisciplinary collaborative approaches level, which may usher in the era of glaucoma therapy based on biomechanical regulation in the future. Our study provides the comprehensive analysis of glaucoma biomechanics research, generating a visualized overview including publication trends, global collaborations, and research hotspots. These findings enable the research community to offer the emerging themes and frontiers, as well as guide future research on glaucoma biomechanics.

## Data Availability

The original contributions presented in the study are included in the article/supplementary material, further inquiries can be directed to the corresponding author.
